# Supplementary figures and images for: Diversity of fall armyworm, Spodoptera frugiperda and their gut bacterial community in Kenya
Source: PeerJ. 2020 Mar 5;8:e8701. doi: 10.7717/peerj.8701 (PMC7060952; doi:10.7717/peerj.8701)

Relative abundance

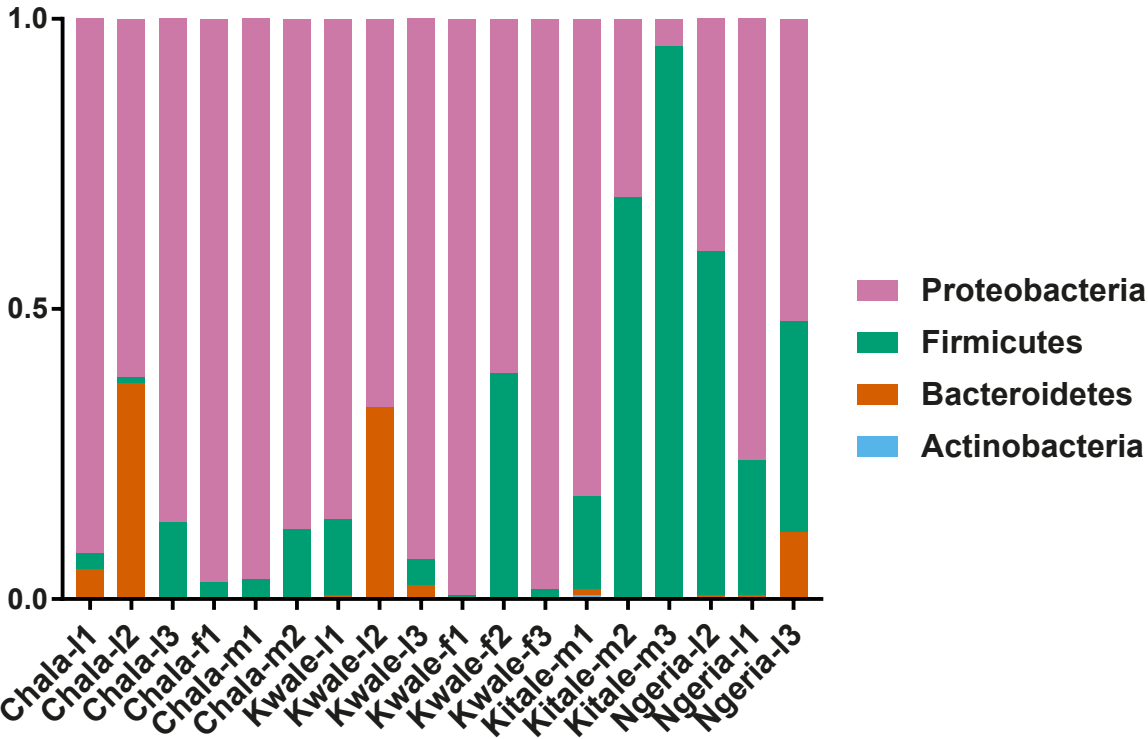

Supplement: Supplemental Information 1 [file peerj-08-8701-s001.pdf]
